# Lucky iPSCs

**DOI:** 10.1186/gb4167

**Published:** 2014-03-25

**Authors:** Asaf Zviran, Jacob H Hanna

**Affiliations:** 1 Department of Molecular Genetics, Weizmann Institute of Science, Rehovot 76100, Israel

## Abstract

The probabilistic behavior of direct induction of pluripotency has been a subject of intense research interest. Here we discuss recently published reports on this topic.

## 

Induced pluripotent stem cells (iPSCs) can be generated from somatic cells by ectopic expression of different pluripotency-promoting transcription factors, canonically Oct4, Sox2, Klf4 and Myc (abbreviated as OSKM) [1]. Exogenous elements involved in signaling and chromatin modification, such as inhibition of ERK and GSK3 signaling (known as ‘2i’ conditions), Lif/Stat3 signaling and/or inclusion of ascorbic acid-containing supplements, directly participate in the reactivation of the endogenous pluripotency circuitry by OSKM [2]. However, OSKM reprogramming is a relatively inefficient process in which only a small minority of somatic donor cells become reprogrammed after an initial period of 10 to 14 days (0.1% to 15% efficiency) [3]. A number of recent studies have sought to better understand the nature of this inefficiency, and how it might be overcome [4-6].

## Characterizing iPSC reprogramming dynamics

**Figure 1:**
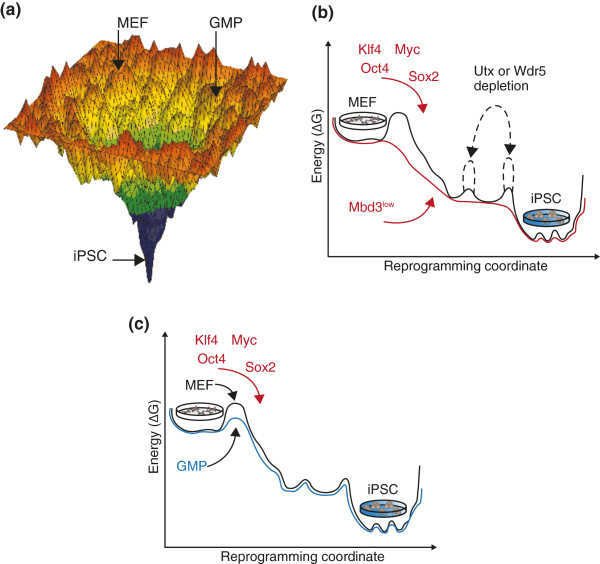
**Energy landscape models for iPSC reprogramming. (a)** Illustration of the energy landscape experienced by cells under reprogramming conditions. The funnel-like energy landscape represents the path from somatic cell states (for example, MEF and GMP) at the boundaries of the funnel, to the pluripotent state at the middle lowest energy point. **(b)** Energy plot of the path from MEF to iPSC under different epigenetic perturbations. The black line represents the energy landscape experienced by wild-type MEFs. The red line represents the energy landscape experienced by Mbd3-depleted (Mbd3^low^) MEF donor cells. This depletion reduces energy barriers and accelerates reprogramming by OSKM to a near-deterministic process. Notably, imposed barriers can occur by depletion of non-rate-limiting essential factors, such as Utx and Wdr5 epiegentic reegulators, whose depletion inhibits and impairs the reactivation of pluripotency genes by OSKM, as marked by the imposed dashed black line barriers [2]. **(c)** Energy plot of the path from MEF (black line) or GMP (blue line) states to the iPSC state. Energy peaks represent barriers in the reprogramming path, where higher barriers correspond with low conversion rates. Different cell types experienced different barrier landscapes [4]. GMP, granulocyte monocyte progenitor; iPSC, induced pluripotent stem cell; MEF, mouse embryonic fibroblast; Mbd3, methyl-CpG-binding domain protein 3; OSKM, Oct4, Sox2, Klf4 and Myc.

A comprehensive investigation of the first 12 days of mouse embryonic fibroblast (MEF) reprogramming by time-lapsed live microscopy allowed the tracking of single early committing founder cells throughout their rapid conversion to iPSCs [7]. This approach highlighted the characteristics of the small fraction of donor cells (up to 3%) that rapidly undergo an early iPSC commitment event, which can occur as early as the first cell division after induction. The clonal reprogramming of the entire cell progeny of these founder cells is detected 7 to 14 days later. Different donor somatic cell types (keratinocytes, pro-B cells or neural progenitor cells, for example) yield different efficiencies for early committing iPSCs after 7 days of OSKM induction (Figure 1a). These differences are possibly due to a variety of determinants, such as cell-type differences in endogenous expression of pluripotency-promoting factors (Sox2 and Klf family members) or reduced tendency for oncogene-induced senescence following OSKM induction.

A more extended period of tracking monoclonal reprogramming dynamics (up to 18 weeks) in pro-/pre-B cell populations that did not yield iPSCs in the first 14 day period, and yet continued to expand, indicated that these populations can eventually give rise to daughter cell iPSCs at unpredicted latency [3]. Importantly, even when starting with homogenous donor cell populations and inducing a relatively more optimized OSKM stoichiometry, whether an individual donor somatic cell will follow an early committed reprogramming trajectory (termed as ‘privileged’ by Guo *et al.*[4] - see next section) or a protracted asynchronized conversion is random and cannot be *a priori* and definitively predicted based on surface markers, cell cycle phase or other identifiable pmeters in the original donor cells [3].

A number of studies have attempted to define patterns of cell surface antigens present in those donor cells committed to a reprogramming trajectory [8]. However, although these markers may increase the probability of identifying committed reprogramming intermediates, they are not a definite guarantee for iPSC progression at the single cell level. Another approach utilized cutting edge methods for single cell gene expression analysis at multiple time points during 1 to 3 weeks of MEF reprogramming, and identified specific endogenous expression hierarchies as predictive markers for increased probability of overcoming the restraining element(s) rendering this process stochastic - and hence of entering the committed hierarchical phase leading to iPSC reprogramming completion [9]. Yet again, although such markers are suitable for identifying cells that have overcome these restraining element(s), they are not necessarily predictive of the probabilistic trajectory assumed at the single donor cell level upon OSKM induction.

A conceptually different approach presented by our group attempted to enable all donor cells to deterministically and inevitably follow a ‘privileged’ and synchronized approach, and from multiple donor cell types, by dismantling epigenetic repressors that hamper the rapid reactivation of endogenous pluripotency genes. Indeed, depletion of the methyl-CpG-binding domain protein 3 (Mbd3) co-repressor coupled with naïve pluripotency conditions (supplemented with 2i/Lif and ascorbic acid) synergistically facilitated up to 100% synchronized iPSC formation from a variety of mouse cells (including MEFs, neural progenitor cells and pro-/mature B cells) following adequate OSKM induction [2] (Figure 1b). This approach also proved successful with *in vitro* differentiated human fibroblast-like cells when applying recently characterized human naïve pluripotency conditions.

A recent study by Guo *et al.*[4] reported ‘privileged’ granulocyte monocyte progenitor cells (GMPs) that exhibit ‘nonstochastic’ reprogramming, where up to 20% of freshly isolated cells retain a highly proliferative state and convert rapidly into iPSCs within 5 to 10 days of OSKM induction. Another study indicated that a short pulse with the transcription factor CCAAT/enhancer-binding protein-α (C/EBPα) in mouse pro-/pre-B cells could lead to their rapid and poised induction to pluripotency by OSKM within up to 8 days in 2i/LIF- and ascorbic acid-containing conditions [5]. But how do these important findings complement and fit into the rapidly evolving framework for reprogramming dynamics and its molecular regulation?

## ‘Privileged’ somatic cells or ‘just lucky’ iPSCs?

In order to set the scene for the discussion of ‘privileged’ somatic cell subpopulations, a simple example can be given. Assuming we ask a population of poker players to draw and reveal their first hand of five cards, some of these players may have a full house as a first hand (despite the low probability of this event). Are they privileged poker players or just lucky?

Guo *et al.*[4] elegantly showed that when challenging freshly isolated and highly proliferative GMPs with OSKM, a subpopulation of 10% to 20% exhibits fast reprogramming with almost uniform progeny conversion to pluripotency (Figure 1a,c). The authors attribute this behavior to the rapid proliferative state of GMPs (8-h cell division rate). They also showed that when these cells were expanded *in vitro* under low proliferating conditions, the percentage of privileged reprogramming-derived cells decreased dramatically from the same somatic cell state. Hematopoietic stem cell progenitors (lineage-negative c-Kit-positive Sca-1-positive (LKS) cells), which are less differentiated than GMPs, reprogram at lower efficiency than freshly isolated GMPs, thus refuting previous claims that the differentiation state *per se* is a critical determinant in reprogramming. Instead, important technical pmeters, such as proliferation rate, can alter the outcome of iPSC reprogramming. It is important to note that privileged reprogramming, where commitment occurs early and randomly after overcoming the main barrier(s), is not restricted to GMPs, and is observed in other somatic cells, such as LKS cells and MEFs, albeit at much lower efficiency [4]. These results corroborate previously published studies on tracking reprogramming early after OSKM induction by live video imaging [7].

Intriguingly, Guo *et al.*[4] used the term ‘nonstochastic’ to describe the ‘privileged’ reprogramming trajectory assumed by GMPs. However, in our opinion, the results presented do not support deterministic behavior at the GMP donor cell population level. In contrast to the spectrum of stochastic behaviors described before, a nonstochastic or deterministic behavior is very stringently defined and dictates synchronized reprogramming of all donor cells and their progeny with a fixed latency immediately following OSKM induction. However, none of the examined cell populations showed nonstochastic or deterministic reprogramming dynamics where near 100% of donor cells had synchronized reprogramming efficiency [4]. Whether a single cell from a GMP population adopted a ‘privileged’ or protracted trajectory remained stochastic, with a 10% to 20% chance of following the privileged route.

Even when Guo *et al.*[4] sorted GMPs into slow- and fast-dividing subpopulations, the latter showed only a slight increase in reprogramming efficiency (from 2% to 6%). The fact that fast-dividing GMPs in general have a higher probability of ‘privileged’ reprogramming than slow dividing GMPs, or other donor cell types, does not render reprogramming by OSKM in these cells nonstochastic because one cannot *a priori* definitively predict the trajectory of an individual GMP cell from the starting purified cell population [2]. The fast dividing GMPs appear to have stochastic reprogramming but with higher probability to commit early to the process, possibly due to the accelerated cell cycle. Indeed, the quantitative change in iPSC reprogramming could be explained by the threefold acceleration of the cell cycle in the fast dividing cells [4].

Importantly, Guo *et al.* tested whether increasing the cell cycle rate of LKS donor cells increased the probability of adopting a ‘privileged’ iPSC reprogramming trajectory. Cytokine-stimulated ultra-fast dividing LKS donor cells acquired monoclonal ‘privileged’ reprogramming efficiency of 3.6% (a greater than tenfold increase relative to slow proliferative conditions). Still, the majority of the donor LKS cell population, even under highly proliferative conditions, did not undergo ‘privileged’ reprogramming following OSKM induction; whether rapid early commitment occurred in a clonal population from rapidly proliferating OSKM-challenged LKS cells was random (with a probability of 3.6%).

## Reprogramming and cell cycle dependency

Increasing the cell cycle by genetic manipulation can accelerate reprogramming dynamics [3]. This acceleration is proportional to the cell cycle change and relates to the increased number of cell divisions during the reprogramming process. Guo *et al.*[4] showed that after 6 days of OSKM induction in MEF cells, a small subpopulation (0.8%) of fast-dividing cells appears that accounts for almost all reprogrammed iPSCs detected early in reprogramming. These results are consistent with the identification of a fast-dividing subpopulation as an early intermediate in the reprogramming commitment [7], which has an increased probability of finishing the conversion to pluripotency. Importantly, however, this fast-dividing subpopulation, characterized as ‘privileged’ [4], is stochastically induced after 6 days of OSKM induction, and is not present in donor MEFs prior to OSKM induction. Hence, these cells can be defined as ‘lucky’ cells that underwent commitment towards iPSCs and assumed the accelerated cell cycle typical of pluripotent cells. However they cannot amount to nonstochasticity, as they are randomly induced after OSKM expression. Further, applying the term ‘privileged somatic cells’ to MEF cells that have undergone OSKM for 6 days is controversial, given that several studies have shown that early iPSCs can already be obtained at this time [2,8].

The authors went on to show that p53 knockdown increases the fast-dividing subpopulation after 6 days of OSKM, allowing for a larger fraction of cells to become fully reprogrammed (1.6%). The authors concluded that a rapid cell cycle state endows the cells with greater competence to assume a ‘privileged’ reprogramming route following OSKM induction. However, these results could alternatively be explained by the twofold cell cycle rate increase caused by p53 depletion, which increases the rate of conversion to the first intermediate of fast dividing cells and so may underlie the twofold increase in reprogramming efficiency observed by Guo *et al.*[4]. In summary, the mechanistic basis for the dependency between reprogramming efficiency and cell cycle rates is not yet understood and constitutes an important direction for further mechanistic research.

## Cell type-specific poising for rapid reprogramming

Di Stefano *et al.*[5] elegantly revisited previous studies that employed overexpression of C/EBPα to boost mouse B-cell reprogramming to iPSCs by OSKM. The authors devised an 18-h pulse of C/EBPα expression followed by OSKM induction in mouse pro-/pre-B cells as a means to achieve rapid reactivation of the Oct4-GFP pluripotency reporter in up to 60% of the donor population within 8 days. When adding 2i and ascorbic acid supplements, the efficiency increased to 95%. C/EBPα expression was accompanied by induction and nuclear translocation of tet methylcytosine dioxygenase 2 (Tet2), which converts methylcytosine to hydroxymethylcytosine, and an increased chromatin accessibility of endogenous pluripotency genes [5].

The above results are intriguing and provide further support for the feasibility to render the reprogramming process near deterministic [2]. Several interesting questions relating to C/EBPα's role remain to be identified. First, its enhancing effect is specific to pre-/pro-B cells - this is absent even in more mature B cells, T-cell progenitors and MEFs [2]. A mechanistic explanation remains to be fully defined, as Tet2 induction is unlikely to be the only cause for the observed dramatic change in reprogramming kinetics, since Tet2 depletion leads to a very mild reduction in iPSC reprogramming of somatic cells by OSKM. Whether the Mbd3/nucleosome remodeling and deacetylase (NuRD) complex or other co-repressor complexes are perturbed following this reprogramming strategy remains to be tested. Finally, extra caution should be taken when conducting and quantifying iPSC reprogramming from hematopoietic cells, as mouse embryonic stem cell culture conditions are not compatible with hematopoietic cell survival and can artificially bias selection for early reprogrammed cells, increasing their relative fraction rather than their absolute number. Indeed, Di Stefano *et al.* indicated that OSKM induction along with their pluripotent reprogramming conditions rapidly depleted nonreprogrammed cells [5]. It may be advisable to routinely exclude cell death and biases in somatic cell survival as the predominant causes for increased reprogramming efficiencies in any future studies, by using more efficient and rapid OKSM inducing animal models, live video imaging and constitutive fluorescence markers for cell survival [2,7]. Nevertheless, the findings by Di Stefano *et al.* present a new means for cell type-specific robust reactivation of the endogenous pluripotency network by OSKM.

## Summarizing thoughts and future perspectives

These recent studies synergistically lead to a model in which the critical rate-limiting factor to reestablishing pluripotency in somatic cells is to rapidly breach through the barrier of reactivating the endogenous pluripotency interconnected circuit (Figure 1). Once this threshold is passed, pluripotency becomes an inevitable outcome and hijacks somatic cell identity [9] (Figure 1b,c). Some cells can achieve this randomly upon OSKM induction with different probabilities [4]. One means to allow nearly all cells to do this early is to reduce epigenetic co-repressors, such as Mbd3, that are recruited by OSKM in a counterproductive manner and hamper pluripotency reactivation [2]. Another strategy is to use cell type-specific boosters, such as C/EBPα, that achieve robust and rapid reactivation of pluripotency by OSKM [5].

Further, these studies highlight the interplay between transcription factor and chromatin changes when inducing the complete epigenetic reprogramming that leads to authentic pluripotency induction. While in OSKM-triggered iPSC reprogramming, OSKM initiate the critical secondary epigenetic events, recent studies have pointed to what may be a global epigenetic change as an initial trigger that can randomly lead to secondary transcriptional reactivation of the endogenous pluripotency network without exogenous transcription factor expression. A prominent example is the demonstration that small molecule compound combinations, several of which target epigenetic repressors, are sufficient to induce the pluripotency transcriptional network in mouse fibroblasts [10]. Another recent sensational study reported the ability to trigger acquisition of pluripotency in neonatal mouse cells by transient exposure to low-pH conditions (with the resulting cells termed ‘STAP’ cells) [6]. If confirmed, these studies may underscore a ‘chicken-or-the-egg’ pdigm regarding the co-dominance between transcription factors and chromatin landscape. Detailed understanding of the interplay between these components is key to the molecular understanding of cell reprogramming and lineage specification trajectories.

## Abbreviations

2i: inhibition of ERK and GSK3; C/EBPα: CCAAT/enhancer-binding protein-α; GFP: green fluorescent protein; GMP: granulocyte monocyte progenitor; iPSC: induced pluripotent stem cell; LKS: lineage-negative c-Kit-positive Sca-1-positive; Mbd3: methyl-CpG-binding domain protein 3; MEF: mouse embryonic fibroblast; NuRD: nucleosome remodeling and deacetylase; OSKM: Oct4, Sox2, Klf4 and Myc; STAP: stimulus-triggered acquisition of pluripotency; Tet2: tet methylcytosine dioxygenase 2.

## Competing interests

The authors declare that they have no competing interests.
